# Hypothalamic Ventricular Ependymal Thyroid Hormone Deiodinases Are an Important Element of Circannual Timing in the Siberian Hamster (*Phodopus sungorus*)

**DOI:** 10.1371/journal.pone.0062003

**Published:** 2013-04-18

**Authors:** Annika Herwig, Emmely M. de Vries, Matei Bolborea, Dana Wilson, Julian G. Mercer, Francis J. P. Ebling, Peter J. Morgan, Perry Barrett

**Affiliations:** 1 Rowett Institute for Nutrition and Health, University of Aberdeen, Bucksburn, Aberdeen, United Kingdom; 2 School of Biomedical Sciences, University of Nottingham Medical School, Queen Medical Centre, Nottingham, United Kingdom; Kent State University, United States of America

## Abstract

Exposure to short days (SD) induces profound changes in the physiology and behaviour of Siberian hamsters, including gonadal regression and up to 30% loss in body weight. In a continuous SD environment after approximately 20 weeks, Siberian hamsters spontaneously revert to a long day (LD) phenotype, a phenomenon referred to as the photorefractory response. Previously we have identified a number of genes that are regulated by short photoperiod in the neuropil and ventricular ependymal (VE) cells of the hypothalamus, although their importance and contribution to photoperiod induced physiology is unclear. In this refractory model we hypothesised that the return to LD physiology involves reversal of SD expression levels of key hypothalamic genes to their LD values and thereby implicate genes required for LD physiology. Male Siberian hamsters were kept in either LD or SD for up to 39 weeks during which time SD hamster body weight decreased before increasing, after more than 20 weeks, back to LD values. Brain tissue was collected between 14 and 39 weeks for *in situ* hybridization to determine hypothalamic gene expression. In VE cells lining the third ventricle, expression of *nestin*, *vimentin*, *Crbp1* and *Gpr50* were down-regulated at 18 weeks in SD photoperiod, but expression was not restored to the LD level in photorefractory hamsters. *Dio2, Mct8* and *Tsh-r* expression were altered by SD photoperiod and were fully restored, or even exceeded values found in LD hamsters in the refractory state. In hypothalamic nuclei, expression of *Srif* and *Mc3r* mRNAs was altered at 18 weeks in SD, but were similar to LD expression values in photorefractory hamsters. We conclude that in refractory hamsters not all VE cell functions are required to establish LD physiology. However, thyroid hormone signalling from ependymal cells and reversal of neuronal gene expression appear to be essential for the SD refractory response.

## Introduction

The Siberian hamster’s natural habitat ranges from the Steppes of Kazakhstan to Mongolia and southern Siberia. This region encompasses a temperate and boreal zone with seasonal changes in climate and photoperiod. Consequently the Siberian hamster can experience a seasonal reduction of temperature to as low as −72°C during the winter months and a concomitant reduction in food availability. To survive and thrive with these seasonal climatic differences, the Siberian hamster adapts with anticipatory physiological and behavioral adjustments. Body weight and reproduction peak during the long days of late spring-early summer, whereas appetite and body weight are reduced and reproduction ceases during the shorter days of autumn and early winter [1], [2]. These adaptations are driven by the annual cycle in photoperiod and can be induced in the artificial environment of the laboratory by changing photoperiod from a long day length (LD) to a short day length (SD). Within approximately 12–16 weeks in a SD environment, physiological changes are complete but can be reversed by switching hamsters back to LD. Spontaneous reversal of physiology and behavior will also occur if Siberian hamsters are held in SD for a period of more than 20 weeks [3]–[8]. This phenomenon is referred to as the photorefractory response and maybe regarded as a manifestation of an innate long-term (circannual) timing mechanism. Similar to robustly circannual species such as sheep and hibernating mammals [9], [10], this timing mechanism enables the Siberian hamster to adopt the appropriate physiology and behavior in anticipation of spring and the forthcoming breeding season. However, the mechanism underlying long term or circannual timing is not known.

In non-hibernating mammalian species, photoperiod control over neuroendocrine pathways is mediated by the hormone melatonin, produced and secreted from the pineal gland during the hours of darkness [11]. The principal site of melatonin action is thought to be the pars tuberalis (PT) of the pituitary gland. The key hormonal output of the PT is thyroid stimulating hormone (TSH) which is released into the median eminence during LD where it acts at the TSH receptor (*Tsh-r*) on neighbouring ventricular ependymal (VE) cells lining the third ventricle of the hypothalamus. Thereafter, in a generalised mechanism which also applies to avian species, TSH stimulates an increase in the expression of type 2 deiodinase (*Dio2*) enzyme in VE cells (largely thought to consist of tanycytes) leading to an increase in the synthesis and supply of tri-iodothyronine (T3) to hypothalamic neurons [12]–[14]. In SD in the mammalian system melatonin suppresses TSH production by the PT leading to a reduction in TSH stimulated T3 synthesis by VE cells [13]. Control of hypothalamic T3 availability is further imparted by type 3 deiodinase (*Dio3*), which catabolises T3 to inactive metabolites [15]. *Dio3* is regulated by photoperiod in VE cells in a reciprocal manner with *Dio2*, although the temporal regulation and the extent to which *Dio2* is down-regulated may be species dependent from modest regulation in Siberian hamsters [16] to robust regulation in sheep [13]. Nevertheless, collectively studies performed in both mammals and birds on the temporal mRNA expression patterns of *Dio2* and *Dio3* together with thyroid hormone implant experiments in Siberian hamsters provide good evidence to support T3 availability as the principal mediator of long term changes in hypothalamic gene expression and subsequent changes in physiology [13]–[22]. A similar mechanism operates in birds but in contrast to mammals, TSH expression in the PT is not regulated by melatonin but light [14], [17]–[19], [23].

In addition to components of thyroid hormone metabolism, VE cells express a number of other genes that change transcription with altered photoperiod. These genes include the orphan melatonin-related receptor *Gpr50* [24], [25], the retinol transporter *Crbp1* and the intermediate filament proteins *vimentin* and *nestin* [26], [27]. The role of these genes in VE cell responses to photoperiod and any contribution to the central mechanism underlying seasonal adaptations is unclear.

A further cohort of genes has been identified to be photoperiod responsive in neurons of hypothalamic nuclei of the Siberian hamster including the arcuate nucleus (ARC) and ventromedial nucleus (VMN). These genes, which are likely to be involved in the adaption of physiological responses, include melanocortin 3 receptor (*Mc3r)*, and somatostatin (*Srif),* among others [28]–[30], but as for VE cell components, their role in the seasonal response remains to be determined.

We have previously shown that the levels of expression of a number of both VE cells and neuronal genes established in SD hamsters can be reversed when hamsters are switched from SD to LD concomitant with a reversal to LD physiology [31]. However, in SD refractory hamsters some genes investigated did not change back to the LD state, indicating a potential difference in the mechanism underlying the reversal of physiology by changing the photoperiod regime compared to the innate timing mechanism that underpins the long-term timing system.

To probe further into the mechanism responsible for the restoration of LD physiology, we investigated photo-responsive gene expression patterns in the hypothalamus of hamsters held in SD for up to 39 weeks. We hypothesized that genes which are important in the establishment of LD physiology in the SD refractory state would be restored to LD expression levels. Emphasis was placed on VE cells, in particular the potential for T3 produced in VE cells to act as a possible driver for changes in hypothalamic gene expression. The data indicate that neuronal changes are important to physiological adaptations in the refractory state. Increased VE cell *Dio2 *mRNA expression in refractory hamsters supports the view that T3 is the likely driver of gene expression change. However, in the absence of a reversal of many of the SD induced mRNA expression changes in the VE cell layer of refractory hamsters, other functions of these cells may not be required for physiological adaptations in the refractory state.

## Materials and Methods

### Ethics statement

Male 3 month old Siberian hamsters, bred at the University of Aberdeen, Rowett Institute for Nutrition and Health, were used for these experiments. All animal work was licensed under the Animals (Scientific Procedures) Act of 1986 and approved by the University of Aberdeen, Rowett Institute for Nutrition and Health ethics committee. Throughout the entire experiment food and water were available *ad libitum* and rooms were maintained at 22°C.

#### Animals

In all experiments hamsters were individually housed in either long days (LD; 16h light and 8h darkness) or short days (SD; 8h light and 16h darkness). All hamsters used in these experiments were approximately 3 months old at the beginning of the photoperiod studies. At the time of killing these hamsters to collect the brains for *in situ* hybridization, the hamsters were older by the length of time spent in photoperiod. All hamsters in SD responded with a weight loss. Two hamsters in the refractory group of experiment 2 did not show weight gain by week 39 in SD and were therefore removed from the study.

### Experiment 1

Siberian hamsters were kept in either LD or SD and body weight was monitored weekly. After 18 weeks (LD n = 6, SD n = 6), 25 weeks (LD n = 6, SD n = 7) or 37 weeks (LD n = 5, SD n = 5) in their respective photoperiod, hamsters were culled at ZT3 (3h after lights on) by cervical dislocation, brains were dissected, frozen on dry ice and stored at –70°C until required.

### Experiment 2

A second refractory experiment was established which included a cohort of hamsters that were switched at their body weight nadir in SD, back to LD photoperiod. This established a cohort of hamsters for comparison in which the long duration melatonin dependent physiological changes are reversed [31]. Siberian hamsters were kept in LD or SD for 14 weeks (LD n = 6, SD n = 6) or 39 weeks (LD n = 5, SD n = 13) with weekly body weight measurements before being culled at ZT3 by cervical dislocation. A third SD group (n = 10) was switched back to LD after 16 weeks in SD and culled (at ZT3) alongside an LD control group (n = 6) at week 22 of the experiment. All brains were dissected, frozen on dry ice and stored at –70°C until required.

### Generation of riboprobes

Riboprobes were generated from DNA fragments for *Dio2*, *Dio3*, *Mct8, nestin*, *vimentin*, *Crbp1*, *Gpr50*, *Mc3r and Srif*, as previously described [16], [21], [26], [28], [32]. A probe for the *Tsh*-r was cloned from mouse hypothalamic cDNA with the following primers based on Genbank sequence NM_011648 – forward primer (bases 607–628) 5′ TCCAGGGMCTATGCAATGAAAC and reverse primer (bases 918–897) 5′ CAGCCCGAGTGAGGTGGAGGAA. A probe for *vimentin* was cloned from hamster hypothalamic cDNA with the following primers based on the mouse *vimentin* sequence (NM_011701; bases 780–794 and 1291–1270) forward primer 5′ AGAACACCCGCACCAACGAGAAGG and reverse primer 5′ ACGCAGGGCAGCRGTGAGGTC. ^35^S riboprobes were generated from 0.5–1.0 µg linearized plasmid DNA or 50–100 ng of an amplified insert using T7, T3 or SP6 RNA polymerase as appropriate for transcription of antisense riboprobes.

### Radioactive *in situ* hybridization

The frozen brains were cut (14 µm) in the region spanning the hypothalamus between Bregma –0.10 to –2.54 mm according to the Mouse Brain Atlas of Franklin & Paxinos 1997 and sections were mounted onto poly-L-lysine-coated slides. *In situ* hybridization was carried out as described previously [17]. Briefly, sections were fixed in 4% paraformaldehyde in 0.1 M phosphate buffer (PB), washed in 0.1 M PB, acetylated in 0.25% acetic anhydrate in 0.1 M triethanolamine, and washed again in PB. Sections were dehydrated using graded ethanol. Radioactive probes were applied to the slides in 70 µl hybridization mixture (0.3 M NaCl, 10 mM Tris-HCL (pH 8), 1 mM EDTA, 0.05% transfer RNA, 10 mM dithiothreitol, 0.02% Ficoll, 0.02% polyvinylpyrrolidone, 0.02% BSA and 10% dextran sulphate) and hybridized overnight at 58°C. Post-hybridization, slides were rinsed in 4x SSC and treated with ribonuclease A (20 µg/µl) at 37°C before being washed in decreasing concentrations of SSC and dehydrated using graded ethanol. Slides were dried and apposed to Kodak Biomax MR film for various lengths of time.

In Experiment 1 there was a restricted availability of slides with sections of the 25 week time point, therefore expression of some genes could only be determined at the 18 wk and 37 wk time points.

### Image analysis

Autoradiographic films were scanned at 600 dpi on an Epson scanner linked to a computer running Image-Pro PLUS version 4.1.0.0 analysis software (Media Cybernetics, Wokingham, USA). Integrated optical density was obtained by reference to the ^14^C microscale. *Dio2*, *Dio3*, *Mct8*, *Tsh-r*, *Gpr50*, *Crbp1*, *nestin* and *vimentin* mRNA expression was measured in three to four sections containing the VE cell layer of the 3^rd^ ventricle. *Srif* and *Mc3r* mRNAs were quantified bilaterally from 3 sections containing the ARC. Values were averaged for each animal. Relative mRNA abundance values were calculated by defining LD 18 weeks (experiment 1) or LD 14 (experiment 2) as 100% expression value.

### Statistical analysis

Data were analysed by two-way ANOVA and followed by Tukey test post-hoc when a significant interaction between the two factors (photoperiod/time) was found. When no interaction was observed, a t-test or Mann-Whitney Rank sum test was performed as appropriate for every time point to reveal differences between photoperiod treatment. T-tests were used for the analysis of Dio2 expression in experiment 2 since the photoperiod treatment of the switchback group differed from the other 2 groups. SigmaPlot/Stat 11.0 software (Jandel, California, USA) was used for all analyses. The results are presented as mean +/− SEM and p<0.05 was considered statistically significant.

## Results

### Experiment 1

#### Body weights

**Figure 1:**
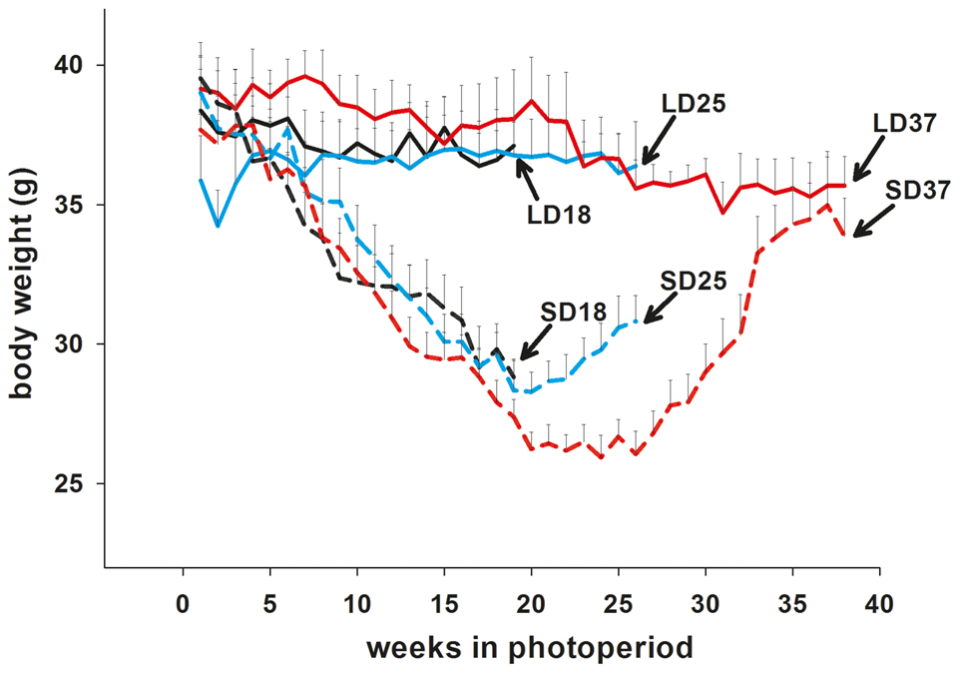
First refractory experiment body weight profiles. Body weights of Siberian hamsters that were kept in long days (LD) or short days (SD) for 18 weeks (LD18 black line, SD18 black dashed line), 25 weeks (LD25 blue line, SD25 blue dashed line), or 37 weeks (LD37 red line, SD37 red dashed line). Data are expressed as mean±SEM.

Hamsters exposed to SD decreased body weight, reaching a nadir between week 20 and 25 (Fig. 1). A two-way ANOVA revealed a significant interaction between photoperiod and time (F = 3.984; p = 0.030). Post-hoc test was carried out which showed within the LD group body weight remained unchanged, whereas a significant increase occurred within SD group between week 18 and week 37 (Tukey p = 0.011). Comparison within week 18 revealed a significant body weight reduction in SD hamsters compared to LD controls (Tukey p = 0.001). This difference between LD and SD animals was still present after 25 weeks (Tukey p = 0.001), although SD hamsters had started to gradually increase their body weight between week 20 and 25. By week 25 in photoperiod, about half of the SD hamsters had regained more than 25% of the total SD body weight loss, but were still significantly lighter than LD controls (P<0.001). This cohort of hamsters constituted a group to analyse gene expression changes during the course of restoration to LD physiology. By 37 weeks in SD, hamsters had reached body weights comparable to LD controls (Fig. 1).

##### Gene expression in ventricular ependymal cells by in situ hybridization (Figs 2–4; Table 1)

Probably as a result of photoperiod induced changes in gene expression being established by 18 weeks in SD, no significant interaction between time and photoperiod was found by two-way ANOVA for the expression of *vimentin* (F = 0.028, p = 0.972), *nestin* (F = 0.366, p = 0.553), *crbp1* (F = 2.47, p = 0.102), *Gpr50* (F = 0.105, p = 0.900) and *Dio2* (F = 1.845, p = 0.176) mRNAs.

**Figure 2:**
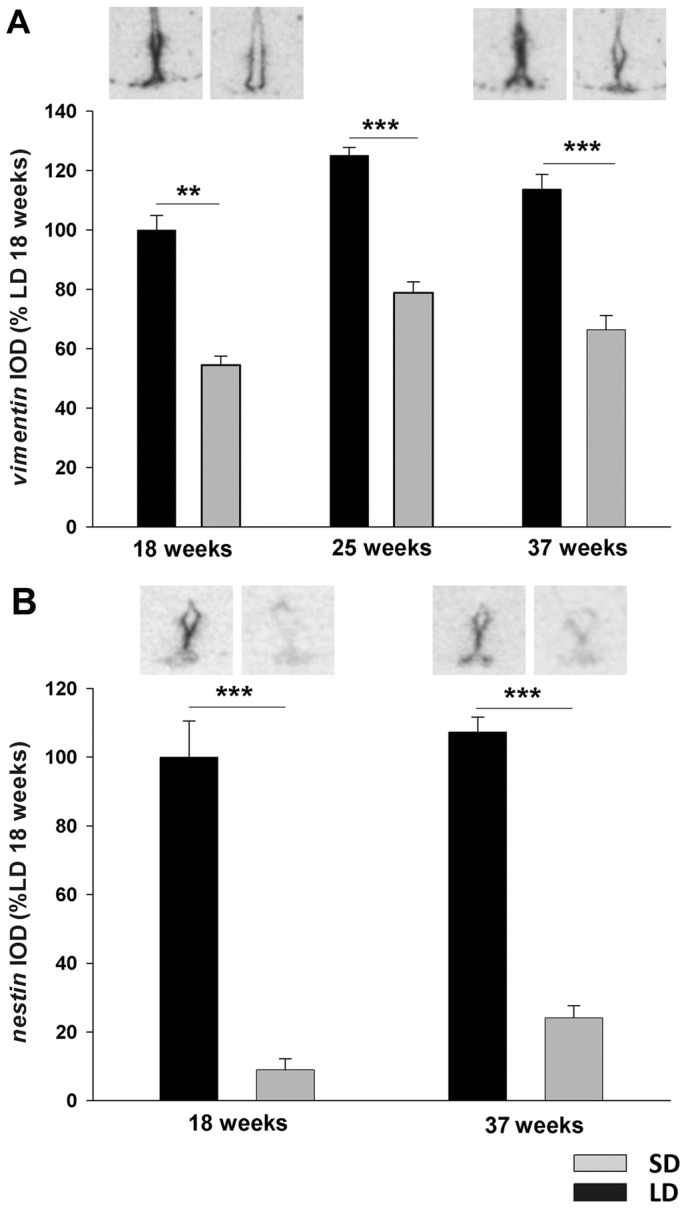
Vimentin and nestin gene expression in ventricular ependymal cells. Autoradiographs and analysis of (A) *vimentin* and (B) *nestin* gene expression in ventricular ependymal cells by *in situ* hybridisation. Siberian hamsters were placed in LD or SD photoperiod for 18, 25 or 37 weeks. Expression was normalized to LD 18 week hamsters. Data are presented as means ± SEM. ***p<0.001

Comparison of integrated optical density for each time point revealed a significant decrease of *vimentin* mRNA in the VE cells (Fig. 2A) in hamsters exposed to SD for 18 weeks compared to LD hamsters (Mann-Whitney_p = 0.002) and continued to remain low in hamsters exposed to SD for 25 weeks (t-test p<0.001) and 37 weeks (t-test p<0.001).

*Nestin* mRNA (Fig. 2B) was determined after 18 and 37 weeks in photoperiod. At both time points, expression was 80% lower in hamsters kept in SD compared to LD hamsters (18 weeks t-test p<0.001; 37 weeks t-test p<0.001).

**Figure 3:**
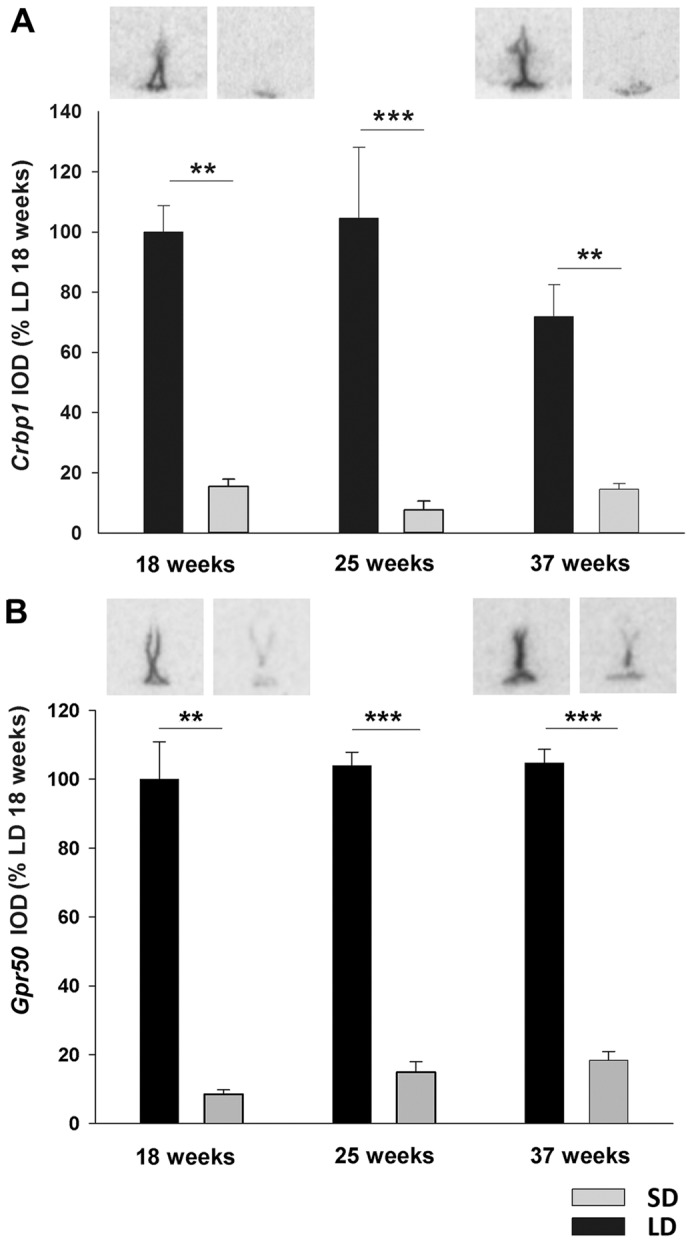
Cellular retinol binding protein and orphan G-protein coupled receptor GPR50 expression in ventricular ependymal cells. Quantification of (A) *Crbp1* and (B) *Gpr50* expression in ventricular ependymal cells of Siberian hamsters that had been kept in LD or SD photoperiod for 18, 25 or 37 weeks. Representative sections are shown in the upper panel. Expression was normalized to LD 18 week hamsters. Data are presented as means ± SEM. ***p<0.001.

Retinoic acid transporter *Crbp1* mRNA expression (Fig. 3A) was decreased by 80% after 18 weeks in SD (Mann-Whitney p = 0.002) and remained at these low expression levels throughout the entire experiment (week 25 Mann-Whitney p<0.001, week 37 Mann-Whitney p = 0.008). Similarly, mRNA expression of the orphan G protein-coupled receptor *Gpr50* (Fig. 3B) had significantly decreased by 18 weeks in SD (Mann-Whitney p = 0.002) and remained 80–90% lower than LD hamsters at all time points (week 25 Mann-Whitney p<0.001, week 37 t-test p<0.001).

**Figure 4:**
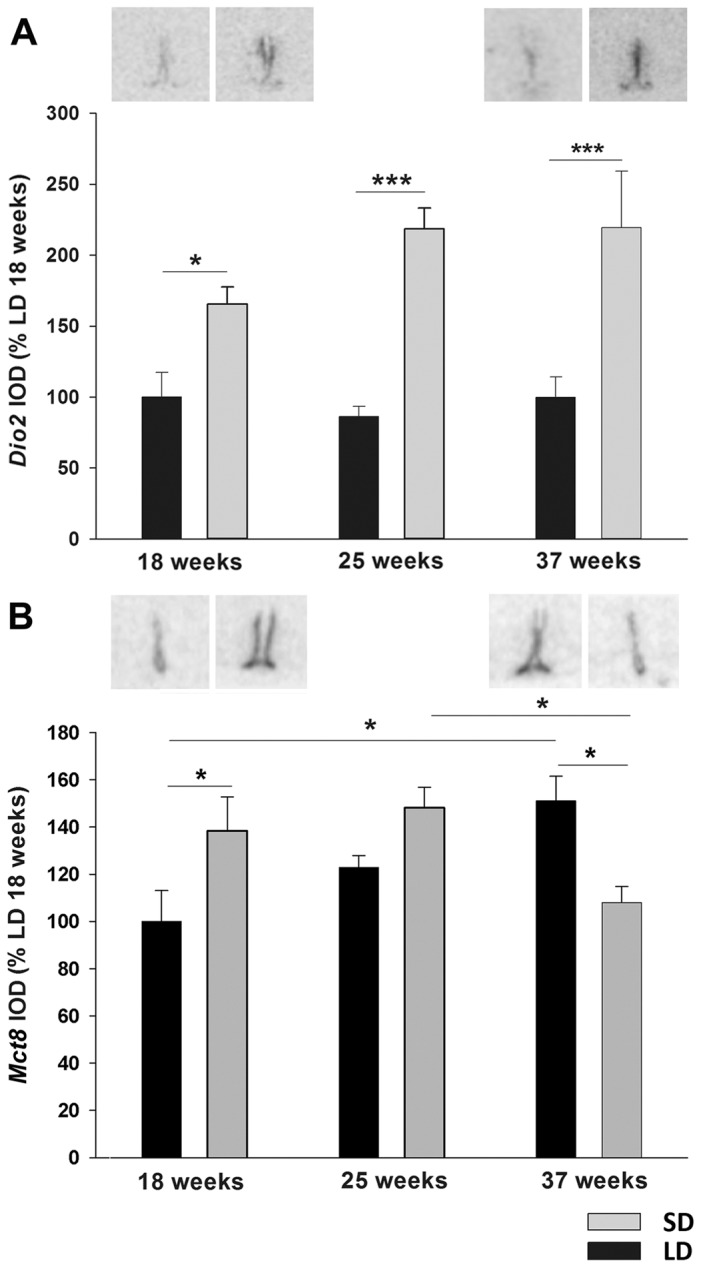
Type 2 deiodinase and thyroid hormone transporter MCT8 expression in ventricular ependymal cells. Analysis and representative sections of (A) *Dio2* and (B) *Mct8* expression in the ependymal cells of Siberian hamsters that had been kept in LD or SD photoperiod for 18, 25 or 37 weeks. Expression was normalized to LD 18 week hamsters. Data are presented as means ± SEM and significance was defined as *p<0.05 and ***p<0.001.

In contrast, *Dio2* mRNA expression (Fig. 4A) was increased by 60% in SD hamsters at 18 weeks t-test p = 0.011), by 130% at 25 weeks (t-test p<0.001) and 37 weeks (t-test p<0.001) relative to the respective LD control groups.

Two-way ANOVA demonstrated significant interaction of time and photoperiod on expression of thyroid hormone transporter *Mct8* mRNA (F = 8.092, p = 0.002) (Fig. 4B). Within LD hamsters there was a significant increase of *Mct8* over time (week 18 vs week 37, Tukey p = 0.006) indicating an age dependent effect. In SD hamsters mRNA levels decreased between week 25 and 37 (Tukey p = 0.028). Within week 18, *Mct8* expression increased 40% in SD compared to LD hamsters (Tukey, p = 0.013). This difference was abolished after 25 weeks (Tukey p = 0.081) and expression levels were decreased relative to LD control after 37 weeks in SDs (Tukey p = 0.011).

##### Gene expression in hypothalamic neurons by in situ hybridization (Fig 5; Table 1)

**Figure 5:**
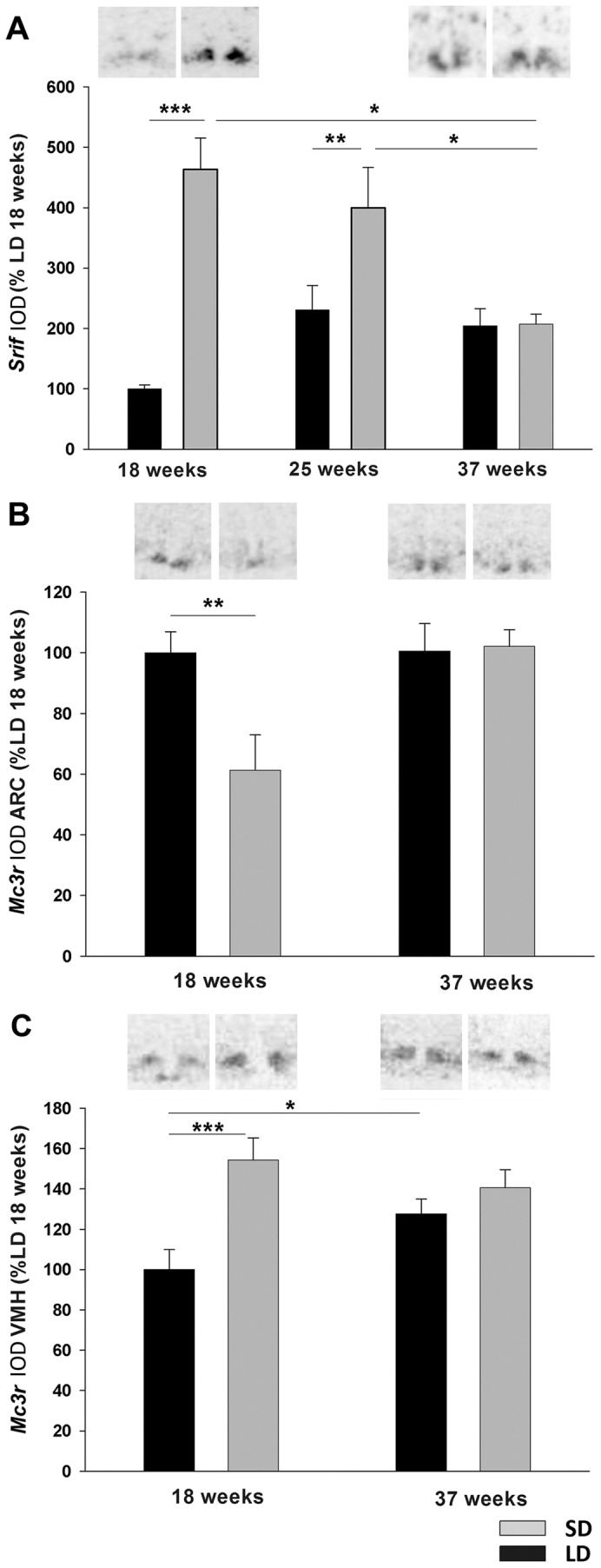
Somatostatin and Melanocortin 3 receptor gene expression in hypothalamus. Analysis and representative sections of (A) *Srif* expression and (B) *Mc3r* expression in the arcuate nucleus (ARC) and (C) *Mc3r* expression in the ventromedial hypothalamus (VMH) of Siberian hamsters that had been kept in LD or SD photoperiod for 18, 25 or 37 weeks. Data are presented as means ± SEM. **p<0.01 and ***p<0.001.

Time and photoperiod showed a significant interaction on *Srif* mRNA expression in the ARC (F = 8.464, p = 0.001) (Fig. 5A). Within SD *Srif* expression was significantly decreased after 37 weeks in SD (week 18 vs week 37, Tukey p = 0.002; week 25 vs week 37 Tukey p = 0.015). At week 18 *Srif* expression was increased by 380% in hamsters that were exposed to SD compared to LD (Tukey p<0.001). This effect was less pronounced but still significant after 25 weeks (Tukey p = 0.003). However, after 37 weeks in SD, *Srif* mRNA expression had returned to LD levels (Tukey p = 0.735).

*Mc3r* mRNA expression was investigated in the ARC and VMH after 18 and 37 weeks in SD photoperiod. In the ARC there was no interaction between time and photoperiod (two-way ANOVA (F = 0.243, p = 0.786) (Fig. 5B). By week 18 *Mc3r* mRNA expression was down-regulated by 40% in the ARC in SD (t-test p = 0.006) but expression had increased to LD levels after 37 weeks (Fig. 5B). In the VMH, time and photoperiod interacted significantly on *Mc3r* expression (F = 5.620, p = 0.030) (Fig. 5C). Within LD *Mc3r* showed a modest increase between week 18 and week 37 (Tukey p = 0.039). After 18 weeks *Mc3r* was increased by 50% in SD relative to LD animals (Tukey p<0.001) but was not significantly different to LD levels by week 37 in SD due to the increase between 18 and 37 weeks in LD hamsters (Fig. 5C).

### Experiment 2

#### Body weights (Fig. 6)

**Figure 6:**
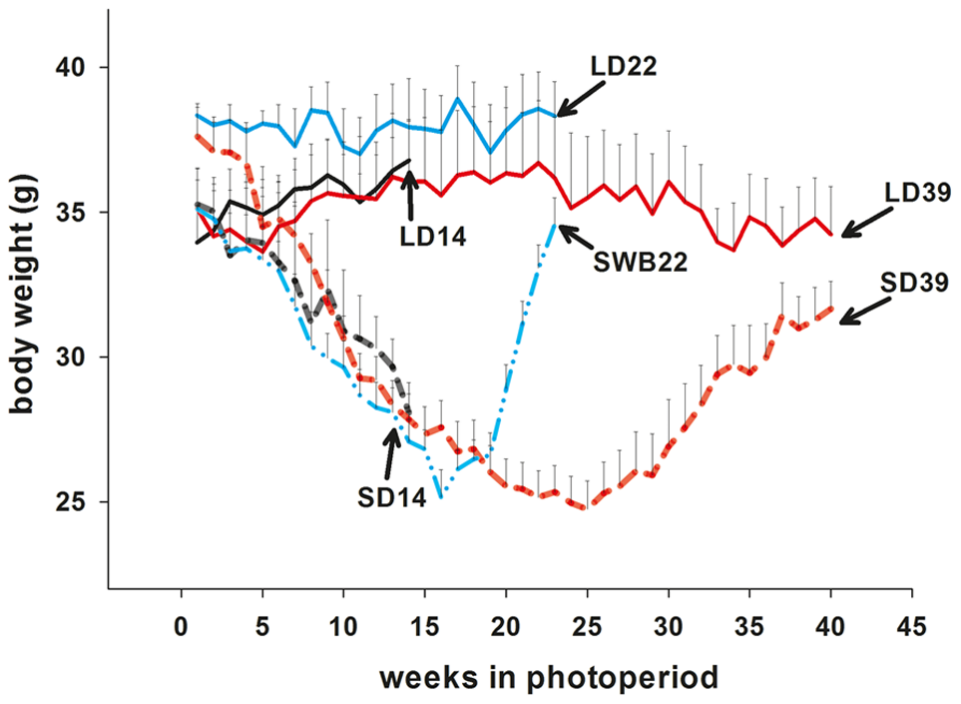
Switchback and second refractory experiment body weight profiles. Body weights of Siberian hamsters that were kept in long days (LD) or short days (SD) for 14 weeks, 39 weeks. After 16 week a group of hamsters was switched from SD to LD (SWB22 blue dashed line) and culled alongside a control group () after 22 weeks. Data are expressed as mean±SEM.

A second refractory experiment was performed in which we also included an additional group of hamsters that were switched after 16 weeks in SD to LD, to induce rapid growth for comparison of the responsiveness of deiodinase gene expression. As in Experiment 1, there was a significant interaction between photoperiod and time on body weights of hamsters (two-way ANOVA F = 3.641, p = 0.035). Hamsters kept in SD decreased and reached a nadir between week 20 and 25 (Fig. 6). After 14 weeks, weight loss in SD hamsters was approximately 80% of maximal and animals weighed significantly less than LD controls (Tukey p<0.001). When a cohort of these hamsters were switched back to LD after 16 weeks in SD, they quickly regained body weight, reaching ∼80% of the weight of their LD controls by week 22 (SWB22, Tukey p = 0.023). After 39 weeks, the remaining hamsters in SD had regained weight and were not significantly different from LD controls.

##### Gene expression changes in ventricular ependymal cells by in situ hybridization (Fig. 7; Table 1)

**Figure 7:**
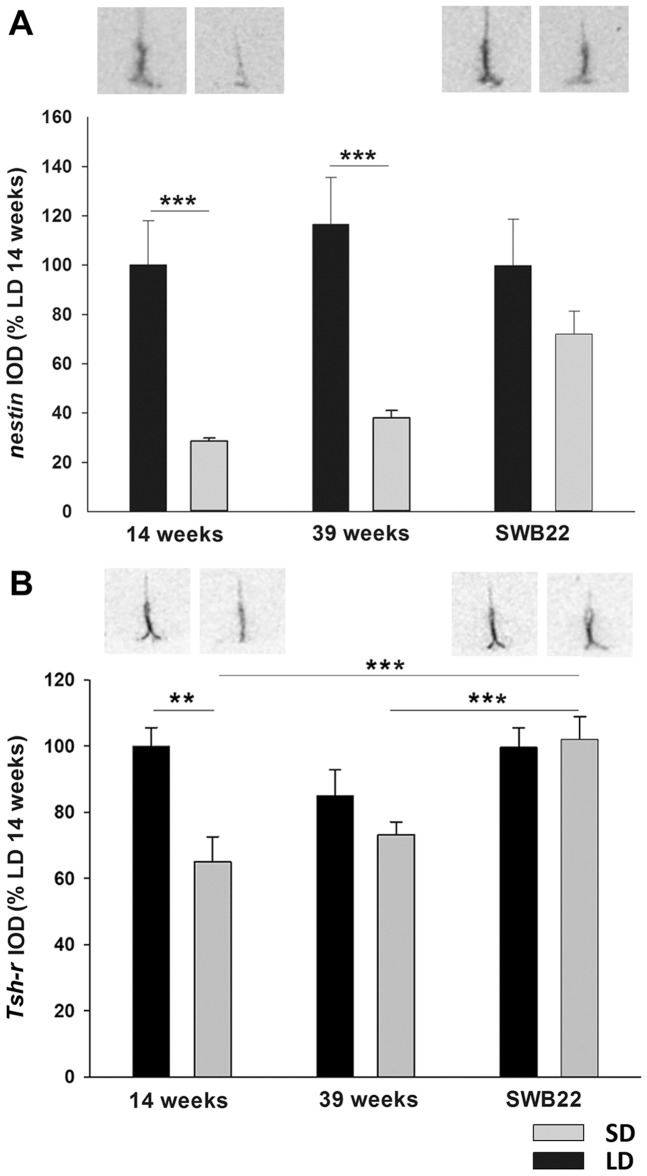
TSH receptor and nestin gene expression in ventricular ependymal cells of switchback and refractory hamsters. Analysis and representative sections of (A) *Tsh-r* and *nestin* (B) in the tanycytes of hamsters that had been kept in LD or SD for 14 weeks or 39 weeks, and hamsters that had been switched from SD to LD after 16 weeks and were culled after 22 weeks (SWB22) alongside an LD control group. Data are presented as means ± SEM and significance was defined as *p<0.05 and ***p<0.001.

An *in situ* hybridization study was performed for *nestin* in this second experiment to both confirm the previous response in refractory hamsters and also to assess the response hamsters switched from SD to LD. As for experiment 1, no interaction of photoperiod and time was revealed for *nestin* mRNA levels (two-way ANOVA F = 3.081, p = 0.057). *Nestin* mRNA expression decreased by 70% after 14 weeks in SD (t-test p<0.001) and remained low after 39 weeks of SD exposure (t-test p<0.001) (Fig. 7A). In the SWB22 group, *nestin* mRNA expression was not significantly different to LD levels.

**Figure 8:**
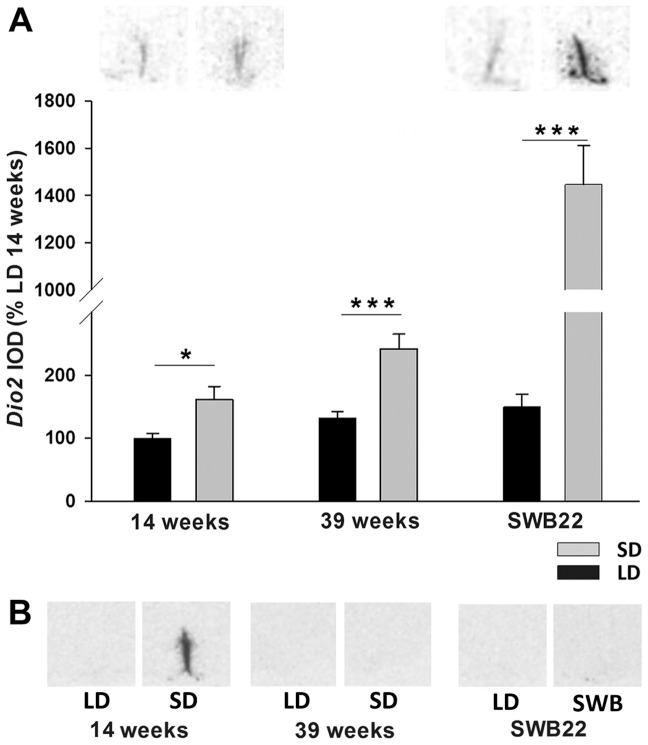
Type 2 and type 3 deiodinase gene expression in ventricular ependymal cells of switchback and refractory hamsters. Analysis and representative sections of (A) *Dio2* and (B) images of *Dio3* expression in the tanycytes of hamsters that had been kept in LD or SD for 14 weeksor 39 weeks, and hamsters that had been switched from SD to LD after 16 weeks and were culled after 22 weeks (SWB22) alongside an LD control group. Data are presented as means ± SEM and significance was defined as *p<0.05 and ***p<0.001. Images shown for *Dio2* expression are from the 14 week and 22 week housed hamsters. Images shown for *Dio3* expression are from the 14, 22 and 39 week housed hamsters.

The TSH receptor is expressed on VE cells and provides the link between the photoperiod dependent signal of TSH production by the PT and *Dio2* mRNA expression in VE cells. Therefore we analysed TSH receptor mRNA expression in VE cells to determine if regulated expression of this gene may contribute to regulation of Dio2 expression in the VE layer. After 14 weeks in photoperiod, there was an interaction of photoperiod and time on *Tsh-r* expression (two-way ANOVA F = 3.721, p = 0.033) (Fig. 8A). Within SD *Tsh-r* mRNA levels were significantly increased in SWB22 hamsters relative to hamsters at 14 and 39 weeks in SD (Tukey p<0.001). At 14 weeks in photoperiod, *Tsh-r* mRNA expression was decreased by 35% in SD hamsters relative to LD hamsters (Tukey p = 0.002), but no difference was apparent after 39 weeks exposure to SD or with the SWB22 group (Fig. 7B).

##### Deiodinase gene expression in VE cells

After 14 weeks in photoperiod, *Dio2* mRNA expression was increased by 60% in SD hamsters (t-test p = 0.023) relative to the LD control group. After 39 weeks in SD, *Dio2* mRNA levels were increased further and were 80% higher than LD controls (t-test p<0.001). However, in the SWB22 group there was a ∼900% increase *Dio2* mRNA expression relative to LD controls (t-test p<0.001) (Fig. 8A).

Significant interaction between photoperiod and time was found for *Dio3* expression (two-way ANOVA F = 10.254, p<0.001). *Dio3* mRNA was absent in LD hamsters and strongly induced after 14 weeks in SD (Tukey p<0.001, Fig. 8B). After 39 weeks in SD or 6 weeks after hamsters were switched from SD to LD (SWB22) *Dio3* mRNA was absent.

## Discussion

Recent studies have shown multiple photoperiod induced changes in gene expression in two contrasting cell populations of the Siberian hamster hypothalamus; ventricular ependymal (VE) cells (which consist primarily of tanycytes) and neurons of the hypothalamus. Many of these photoperiod regulated genes are likely to be involved in the adaptive physiological responses to seasonal changes in climate and participate in a range of processes. Of relevance to VE cells these processes may include thyroid hormone metabolism, retinoic acid signaling, nutrient sensing and cellular plasticity whereas in neurons there may also be a wide variety of processes that vary seasonally including hormone sensing, neurotransmission, cellular activation and cellular secretion [16], [18], [21], [22], [26], [27], [29], [31], [33]–[36]. Precisely which of these genes, their functions and cell type are essential for the establishment of the SD or LD state is not known. Even less is known of the mechanism underpinning the refractory response of seasonal animals which ensures a physiology conducive for survival and reproduction in anticipation of changes in environmental conditions.

The available evidence provides a strong case for the regulation of thyroid hormone availability from VE cells to neighboring hypothalamic neurons as at least one important element in the central regulation of seasonal physiology in response to changing photoperiod. From a mechanistic perspective, the current dogma involves a reduction of thyroid hormone availability to the hypothalamus as a consequence of SD exposure leading to changes in thyroid hormone regulated genes in hypothalamic neurons. In Siberian hamsters the mechanism for initiating SD physiology from the LD state appears to involve an early induction of *Dio3* gene expression in VE cells following a transfer from LD to SD photoperiod and although less clear is likely to involve a reduction in *Dio2* mRNA [15], [18], [30] The rise in *Dio3* enzyme promotes catabolism of T3 and T4 to inactive metabolites leading to a reduced availability of T3 to the hypothalamus [16], [21], [22]. Further supporting the role of T3 in physiological responses to photoperiod, recent findings show that T3-releasing hypothalamic microimplants implanted in hamsters after 11 weeks exposure to SD induce a transition to reproductive and metabolic LD physiology without changing pelage characteristics; this suggests that increasing T3 availability may also underpin the transition from SD to LD physiology [22].

The question therefore arises as to whether an increase in T3 availability could also play a role in the SD refractory response. Our studies reported here provide evidence that a rise in hypothalamic T3 is likely to be involved in the physiological responses of SD refractory hamsters.

At 18 weeks in SD around the nadir of body weight loss, *Dio2* mRNA expression was increased relative to LD hamsters and remained elevated after 25 and 37 weeks in SD concomitant with an increase in body weight. We confirmed this in a second experiment, where *Dio2* mRNA was modestly higher after 14 weeks, but strongly increased after 39 weeks in SD. In the second refractory experiment we also included an additional cohort of hamsters which were transferred to LD after 16 weeks of SD exposure. The switch to LD induced rapid growth returning to LD physiology within 6 weeks. Analysis of *Dio2* mRNA expression in the ventricular wall of these hamsters showed a 900% increase. Although changes in *Dio2* mRNA expression are less robustly affected in response to SD photoperiod than *Dio3* mRNA expression together with our previous studies and those of others [16], [18], [21], [30], [37], the data point to a role of Dio2 enzyme and thyroid hormone availability as a principal components in physiological adaptations in refractory hamsters. While our data is at odds with a previous study in Djungarian hamsters where no increase in *Dio2* expression was reported [37] it is consistent with a recent study on the regulation of deiodinases in a refractory sheep model [38].

The current dogma incorporating evidence from hamsters, sheep, rats and quail on the photoperiod regulation of *Dio2* in VE cells is via photoperiod-dependent regulation of TSHβ production by the PT which acts as a TSHα/β dimer in a paracrine fashion on TSH receptors located on VE cells [12]–[14], [32]. The question therefore arises as to whether TSH or the TSH receptor may be involved in *Dio2* induction in the SD refractory hamster. Evidence provided Bockers et al. would indicate that this is unlikely as expression of the gene for the β-subunit of TSH in the PT remains suppressed although the common glycoprotein hormone α-subunit becomes refractory to the SD melatonin signal [39].

Expression of the TSH receptor in the VE layer was also examined as possible contributor to mediating the signal of photoperiod regulated TSH by the PT to the VE layer. TSH receptor (*Tsh-r*) expression in the VE cell layer was decreased with SD exposure implying reduced receptor number. Expression of *Tsh-r* returned to LD values in hamsters switched from SD to LD, and SD refractory hamsters had similar values to LD hamsters. However, the relevance of this regulation needs further investigation, but has potential to contribute to a photoperiod dependent TSH stimulatory pathway in VE cells. Pertinent to this discussion, we have previously shown that *Dio3* expression is transient with high expression at 8 weeks after transfer of LD hamsters to SD, but thereafter declines over the following weeks until it becomes undetectable sometime after 14 weeks in SD [21]. Consistent with the report of Watanabe et al. [37] we have been unable to detect *Dio3* expression in refractory hamsters and hamsters switched from SD to LD (Fig. 7B). This is further evidence that the temporal order of *Dio3* and *Dio2* expression facilitates the regulation of thyroid hormone availability to the hypothalamus in a time-dependent manner consistent with the appearance of the return to LD physiology. Furthermore evidence for the temporal order of *Dio2* and *Dio3* gene expression in the refractory response has recently emerged from a study on sheep in which the findings are consistent with the findings described here in the Siberian hamster [38].

In addition to regulation of deiodinase mRNA expression, the expression of the thyroid hormone transporter *Mct8* mRNA is induced by SD photoperiod in the VE cell layer [16]. Here we show that the increase in *Mct8* expression at 18 weeks in SD was reduced relative to the LD values in SD refractory hamsters at 37 weeks, during which time there was an age dependent increase in expression in LD hamsters. The relevance of the increase in *Mct8* mRNA expression in LD is unknown, but potentially could contribute to increased hypothalamic T3 levels in the hypothalamus in LD hamsters. Although the direction of change of *Mct8* with photoperiod appears counterintuitive and is not understood, it does serve to illustrate that another component of the thyroid hormone system in VE cells is restored in refractory hamsters supporting the importance of thyroid hormone in determination of physiology.

In contrast to the aforementioned components of the thyroid hormone system, four other genes photoperiodically regulated in the VE cell layer and chosen on the basis of a range of potential functions did not change from their level of expression established by SD photoperiod with longer SD exposure in refractory animals. These include *vimentin* and *nestin* which have been postulated to be involved in morphological adaptation of the VE cell layer with a consequential impact upon neurosecretion of peptides in the median eminence [40]. The absence of changes in expression of these genes may indicate that such changes are not necessary for the establishment of the LD phenotype of SD refractory hamsters. Similarly *Gpr50* expression decreased in SD and remained low in refractory hamsters. This indicates that the function performed by this orphan receptor in the VE cell layer of the LD hamster, and which has been associated with leptin signaling and thermogenesis in mice [34] is not required to establish the LD physiological state.

Consistent with a previous study, *Crbp1* expression was decreased after 18 weeks in SD and remained down-regulated in SD refractory hamsters [31]. Our previous study also showed that gene expression of other components of the retinoic acid response system (RAR and RXR receptors) did not resume LD expression values in the SD refractory hamster [31]. Together these data are consistent with that there being no requirement for increased retinoic acid signalling for the induction of LD physiology in the SD refractory state.

The fundamental components of a coordinated response in physiology are the neurons. In our work we have identified a suite of gene expression changes in different neuronal populations that are likely to be pertinent to seasonal physiology. This includes *Mc3r* that mediates the action of αMSH on food intake and energy expenditure [28]. In this study we found that SD induced changes *Mc3r* in two separate neuronal populations (VMH increased, ARC decreased) are abolished in the SD refractory state, by a reversal in expression in the ARC or by an increase in LD in the VMH. This normalizing of *Mc3r* expression in the VMH may reflect an age dependent increase since the expression is not normalized by a reversal of the SD expression values. However, the significance an age dependent increase in the VMH is not known for hamster physiology. In addition a SD-induced increase in *Srif* expression in the ARC is decreased to LD values in the SD refractory hamster. Although the decrease of *Srif* in SD refractory hamsters cannot be separated from an age dependent effect, this decrease is entirely consistent with the return to a LD phenotype, particularly if *Srif* in the context of photoperiod may be involved in growth related events [30]. These observations coupled with a previous study where we demonstrated that *H3r*, *Vgf* and *Crabp2* expression in the dmpARC is restored to LD values in refractory hamsters, points to the importance of neuronal genes in establishing the physiology of the SD refractory state. The exception to this so far is *RAR* and *RXR* gene expression localized in the dmpARC, as these are not restored to LD expression values in the SD refractory hamsters [31]. We postulate this differential response may be due to regulation of responsive genes by an increased T3 availability, whilst the non-responsive genes are regulated by retinoic acid, which as a result of continued suppression of the retinol transporter in VE cells cannot be increased in neurons of the SD refractory hamster. At this point it is also interesting to consider the findings of Kauffman et al for the requirement of at least a 6 week period of LD exposure of refractory Siberian hamsters in order to regain sensitivity to the SD melatonin signal [41]. We would predict from our studies that this period of LD exposure is required for genes such as *Crbp1*, *vimentin*, *nestin* and *Gpr50* to attain their LD expression levels before sensitivity to SD can be re-established, i.e. a requirement for physiology of tanycytes in the VE layer to be reset, which may require a stimulatory action of TSH on tanycytes to achieve resetting of the mechanism. Supporting this idea we find that hamsters which have been transferred from SD to LD show a 900% increase in *Dio2* gene expression and restoration of *nestin* gene expression to LD values.

A summary of the gene expression changes in response to an extended period of SD exposure is shown in Table 1. Taken together our data demonstrate intriguing changes in the expression of photoresponsive genes in the hypothalamus of SD refractory Siberian hamsters. We provide evidence for an uncoupling between gene expression changes involved in thyroid hormone metabolism and transport and other photoperiod-regulated genes within the VE cells of the third ventricle. These data provide evidence for temporal changes in thyroid hormone availability in the hypothalamus over time in SD, implicating thyroid hormone metabolism in the regulation of seasonal physiology and demonstrating that not all VE cell functions are likely to be required to re-establish LD physiology in the refractory state. Importantly with few exceptions to date, reversal of SD induced changes in neuronal gene expression in the SD refractory state is required for the re-establishment of LD physiology.

**Table 1:** Overview of the genes of neuronal (standard font) and ventricular ependymal cell (bold font) origin and their response to extended SD photoperiod exposure relative to their LD control groups.

Study 1	18 wks	25wks	37wks
***vimentin***	↓	↓	↓
***nestin***	↓↓	n/a	↓↓
***Crbp1***	↓↓	↓↓	↓↓
***Gpr50***	↓↓	↓↓	↓↓
***Dio2***	↑	↑	↑
***Mct8***	↑	↑	↑
*Srif*	↑↑	↑	=
*Mc3r* ARC	↓	n/a	=
*Mc3r* VMH	↑	n/a	=

Study 2	14wks	39wks	SD-LD
***Dio2***	↑	↑	↑↑↑
***Dio3***	↑↑↑	=	=
***nestin***	↓↓	↓↓	=
***Tsh-r***	↓	=	=

Arrows indicates direction and strength of change in gene expression: ↑ small, ↑↑ moderate and ↑↑↑ large increase in gene expression; ↓ small and ↓↓ moderate decrease in gene expression;  =  no change in gene expression; n/a gene expression was not available.

Photorefractoriness is also an important element of the seasonal breeding patterns of birds [42] and may well share commonalties with the mammalian system [23]. However, it would appear from limited studies that the relationship between expression of *Dio2* and *Dio3* with refractory responses is more complex [37] than describe here for the Siberian hamster. It will therefore be of considerable interest to investigate the expression of other genes in the avian brain to ascertain mechanisms and commonalities in the photorefractory response of birds and mammals.

Finally, if we consider the refractory response as a manifestation of a circannual timer, regulation of *Dio2* and *Dio3* expression is likely to be a principal component of a circannual timing mechanism in the hamster and other species [38].
